# Case of Fracture of the Skull

**Published:** 1862-01

**Authors:** O. B. Ormsby

**Affiliations:** Assistant Surgeon of the 18th Regiment, I. V.


ARTICLE III.

### CASE OF FRACTURE OF THE SKULL.

By O. B. ORMSBY, M. D., Assistant Surgeon 18th Reg't I. V.

Reported for the EXAMINER.

I was called at 12 M. of October 18th, to see Michael B——, Irishman, aged 35, brown hair, blue eyes, and apparently a stout healthy man.

Found him entirely insensible; pupils dilated widely, but equally on either side, pulse 100, small and soft, extremities cold, and breathing so irregular as to warrant description. Each inspiration was accomplished by a series of violent sobs, and was succeeded by an expiration very nearly, or quite normal, in character. After breathing in this manner quite hurriedly four or five times, an interval of ten seconds of entire rest succeeded, during which, the patient did not breathe at all. Upon inquiry, I learned that in a street fray, twelve hours previously, he had received a severe blow on the head. The scalp was unbroken, but considerably tumefied, and it was with some difficulty, that fracture and depression of a portion of the left parietal bone was diagnosed. Venesection in both arms was attempted, but meeting with only partial success, no beneficial result was apparent; and as the vital powers of the patient were evidently failing, with the concurrence of Surgeons BURKE, PAINE, DAVIS and STAHL, an operation for his relief was determined upon.

When the scalp corresponding to the apparent seat of injury was exposed by the razor, a purple discoloration was observed extending antero-posteriorly from 2½ to 3 inches, and perhaps ½ inch in width from above downwards, evidently the result of a blow; otherwise, the scalp appeared to be healthy. The operation was commenced by making a crucial incision 2½ inches in length through the integument, aponeurosis of the occipito-frontalis, fascia and cellular tissue to the bone. The flaps were then dissected up, when an examination disclosed a very close fracture running across the space thus exposed, from before backward, with very slight depression of the inferior portion of bone. The trephine was then applied, and a portion of the fracture cut away. A small branch of the temporal artery, which bled so profusely as to impede this part of the operation, was tied.

The trephine used was one of the conical-shaped, made by Tiemann, having the teeth pitched well forward, and was inclined to take too rank a hold on the diploe, but cut the tables very readily. The portion of bone included within the crown was then taken out, disclosing beneath it a clot of dark colored blood lying upon the dura mater. The elevator was applied by Dr. DAVIS, and the depressed bone raised up to its proper level, affording evidently some relief to the patient, whose breathing became somewhat more regular. After waiting a few minutes, all of the clot which could be got at without undue violence was removed through the opening made by the trephine. The patient was then removed to the hospital of the 18th Regiment, and four drops of ol. tiglii administered per anum, and sinapisms placed upon his abdomen and extremities. The wound was washed with warm water, to encourage oozing of blood, and left open. The patient however soon relapsed into his former condition, gradually sank and died at 9 o'clock P. M., of the same day. Autopsy before the coroner's jury seventeen hours after death: Rigor mortis well marked, body looks healthy, wound resembles a fresh cut. With a view to examine the brain, commenced an incision at the mastoid process of the left temporal bone, carried it through the incision made in trephining directly over the crown, to the corresponding point upon the opposite side, and dissected off the scalp anteriorly and posteriorly, exposing the entire upper and lateral regions of the skull. In this dissection, a considerable quantity of extravasated blood was noticed infiltrating the cellular tissue of the scalp, evidently the result of several bruises. A large fracture was very readily discovered extending from left to right, entirely through the coronal suture, dropping behind it in the middle of the right parietal, but again reaching it before its termination at the squamous suture. Crossing this fracture at right angles was another shorter one, extending from an inch anterior to the coronal suture toward the middle of the inferior border of the left parietal. This fracture was curved, approaching nearer the vertex, in the middle of its course than at either extremity. Three small indentations were observed in the frontal bone, close to the anterior inferior angle of the left parietal, from one of which proceeded a small radiated fracture. Another fracture was discovered commencing at the anterior inferior border of the left parietal, extending upward and backward towards its centre, and dividing near the termination of its course. Another small fracture commenced near the middle of this bone, and extended 1¼ inches backward into its body. A fracture half an inch in length passed backwards from the large transverse fracture into the body of the right parietal.

The next step was the removal of the calvarium; upon an examination of the internal table, found it fractured in various directions, corresponding to the fractures of the external table already described. Found also two large discolorations before and to the right of the vertex, produced by effusion of blood into the diploe. Upon the dura mater, covering the right hemisphere, was found a large clot of blood three inches in diameter, one inch in vertical depth in the centre, and weighing seven and one-half ounces. This clot entirely covered the anterior superior portion of the right hemisphere, and encroached to the extent of an inch upon the left. This blood was found to have proceeded from the middle meningeal artery of the right side, which passed through a canal at the anterior inferior angle of the parietal, and at that point was entirely severed. The dura mater was next dissected off; the brain and membranes presented a healthy appearance, no adhesions, no effusion, and the brain presenting only ordinary vascularity. In the walls of the superior longitudinal sinus a small piece of bone $\frac{5}{8}$ of an inch long, and $\frac{1}{8}$ wide was found, but it had evidently been deposited there some time previously. Removing the brain from the skull, found no fracture or disease of the base. Upon examination, the brain was found to be healthy in every part. The skull was of medium thickness, solid, and the sutures well closed. It was alleged upon the witness stand, that this man had received but one blow from a club, and that subsequently he had spoken, and got up and walked a distance of one hundred yards. The principal questions in the legal investigation of this case to be decided, by medical testimony, will be, could one blow have produced the amount and kind of injury here described, and could the subject, afterward, have walked a distance of one hundred yards. The most surprising feature of the case, however, apparent to me, was, that such extensive fractures had been produced without solution of continuity of the scalp. Had the injury been at the base of the skull, it could be readily accounted for, from the fact that fractures of that region are more often produced by falls, where the weight of the body, transmitted through the spinal column is the immediate cause of the mischief, and the scalp is not necessarily bruised at all; but to injure the vertex, momentum must be applied from without, and to render that momentum effectual, it must be more or less concentrated. If sufficiently diffused, however, to destroy the integrity of the bone so largely, still without injury to the scalp, then the shock to the whole mass of the brain must have been very great, and the consequent concussion correspondingly severe. It is, however, perhaps barely possible, that the man might have walked the specified distance after recovering from the concussion, and before the artery had poured out a sufficient quantity of blood to effectually compress the brain, (for the depression of bone was not nearly great enough to produce so severe symptoms.) But could such an injury, by any possibility, have been produced by a single blow?

